# The relationship of invasive and non-invasive respiratory therapy to the risk of developing the Retinopathy of Prematurity (ROP)

**DOI:** 10.1186/1753-6561-9-S7-A2

**Published:** 2015-10-27

**Authors:** Bolshova Alina Sergeevna, Krushelnickii Anatoliy Aleksandrovich, Stepanova Ekaterina Vladimirovna, Degterev Dmitrii Nickolaevich

**Affiliations:** 1 First Moscow State Medical University named after I.M. Sechenov, Moscow, Russia; 2 People’s Friendship University of Russia, Moscow, Russia; 3 Scientific Center of obstetrics, gynaecology and perinatology named after academician V.I. Kulakov, Moscow, Russia

## Background

ROP occurs in 95% of babies weighting less than 1000 g, all cases that had proceeded to the third stage will cause serious complications or complete blindness [1]. The aim of the study was to evaluate the degree of invasive and noninvasive respiratory therapy affection on the frequency and severity of ROP in the group of risk.

## Methods

Retrospective analysis of examination of 217 newborn of the risk group. ( In Russian Federation all children under 35 weeks of gestation age or weighting below 2000 gr lie in the group of risk for ROP). Due to the development of Respiratory Distress Syndrome all children needed respiratory therapy on the first week of living. The patients were divided into 3 groups according to the kind of respiratory therapy received. 1st group ( n-=86) received noninvasive respiratory therapy with Nosal Continuous Airway Pressure, 2nd group (n=69) – noninvasive therapy with Biphasic, and 3rd group (n=62) – invasive respiratory therapy with Artificial Respirating Unit.

## Results

In 1st group we observed 0 cases of ROP, in 2nd - 3 cases were diagnosed, but all of them had a fast regression afterwards, in 3rd group – 20 children developed ROP, only 15 cases regressed and one child had to go through the laser coagulation of the retina vessels.

## Conclusions

Changing from invasive to noninvasive forms of respiratory therapy may lead to significant decrease of the risk of ROP.
